# Quantification of Caffeoylquinic Acids in Coffee Brews by HPLC-DAD

**DOI:** 10.1155/2014/965353

**Published:** 2014-12-21

**Authors:** Marzieh Moeenfard, Lígia Rocha, Arminda Alves

**Affiliations:** LEPABE, Faculdade de Engenharia, Universidade do Porto, Rua Dr. Roberto Frias, 4200-465 Porto, Portugal

## Abstract

The influence of different brewing conditions on the concentration of the main caffeoylquinic acids (3-caffeoylquinic acid (3-CQA), 4-caffeoylquinic acid (4-CQA), and 5-caffeoylquinic acid (5-CQA)) was investigated. For this purpose, twenty-four coffee brews were extracted and analyzed using HPLC-DAD at 325 nm. Our findings demonstrate the great impact of brewing techniques on the caffeoylquinic acids (CQAs) content. The major isomer was 3-CQA, accounting for about 50% of the total CQAs, followed by 5-CQA and 4-CQA, accounting for about 24–36% for each one. The total content of CQAs was in the range of 45.79 to 1662.01 mg/L, found in iced cappuccino and pod espresso, respectively. In conclusion, this study demonstrates that coffee brews, in particular those prepared using pressurized methods, can be considered as the potential sources of antioxidants such as CQAs.

## 1. Introduction

Coffee is one of the most commercialized food products and may be prepared by different techniques depending on the consumers' preference. From a chemical point of view, the two main coffee species (Arabica and Robusta) may be a rich source of biologically active compounds and their potential human health effects depend on consumers' physiology and the amount of coffee consumed per day [1]. Among several compounds present in coffee, chlorogenic acids (CGAs) are one of the most important groups. Although with considerable variation, total CGAs may account for 7.0–14.4% of dry matter basis in green Robusta and 4.0–8.4% in green Arabica beans [2].

Caffeic, ferulic, and* p*-coumaric acids are the main phenolic compounds in coffee which derive from* trans*-cinnamic acid. Naturally, they may present as mono- or diesters with quinic acid, forming chlorogenic acids [2], which are known to be the most active antioxidant compounds [3]. CGAs are water soluble compounds [4, 5] and they can be divided into caffeoylquinic acids (CQAs) with 3 isomers (3-, 4-, and 5-CQA), feruloylquinic acids (FQA) with 3 isomers (3-, 4-, and 5-FQA), dicaffeoylquinic acids (diCQAs) with 3 isomers (3,4-diCQA; 3,5-diCQA; 4,5-diCQA), and, to a lesser extent,* p*-coumaroylquinic acids (*p*CoQAs) with 3 isomers (3-, 4-, and 5-*p*CoQA). Among them, CQAs are found to be the most abundant compounds in coffee [2, 6]. Since CGAs contribute to acidity, astringency, and bitterness of the brewed coffee [4], they are relevant to sensorial properties of the beverage. Besides that, the antioxidant properties of CGAs are well documented in the literatures [7, 8]. These compounds also possess protective effects against type 2 diabetes and Alzheimer's disease [9].

Literature survey revealed that numerous production steps involved in coffee production may influence the CGAs content in the final product; however, the roasting process is described as the most important step which has a profound effect on chemical composition of the products [10–12]. The CGAs content in brewed coffee may differ according to other parameters, like coffee species [11, 12], origin of beans [13], and subsequent brewing methods [14, 15].

Although the CQAs content in coffee beans and the effect of processing conditions, especially roasting, on CGAs have been widely reported [11, 12, 15], data related to the new brewing procedures are limited [16, 17]. Coffee can be brewed in many ways depending on consumers' preference but recently consumer choices for a particular type of coffee beverage have been affected by various parameters.

Although data indicates that coffee brews are capable of delivering different levels of CQAs (26.1–295.6 mg/100 mL) [15], there is limited information regarding the influence of brewing conditions on the level of CQAs, especially through the new brewing techniques like capsules [16], pods, or easy drinking beverages such as iced coffee. Considering the significant consumption of coffee beverages among European countries and due to the contribution of CQAs as the most important class of CGAs to human health, a comprehensive study was performed to evaluate the effect of wide range of brewing techniques on CQAs content (3-CQA, 5-CQA, and 4-CQA), prepared by various technologies. This would allow us to estimate the role of brewing techniques and the composition of coffee blends in CQAs content of coffee brews and subsequently in equilibrating the acidity of brews for consumers who suffer from acid reflux symptoms. However, some care should be taken into account because some differences regarding nomenclature of 3-CQA and 5-CQA seem to appear in several publications.

Besides that, few research papers reported the validation of the analytical methods with regard to CQAs in the new brewing processes [18].

Therefore, the aim of this work was to evaluate the CQAs content and profile in different brewing processes, including homemade brews (boiled, filter, French, and mocha coffee prepared using economically important coffee species, Arabica and Robusta) and commercial brewed coffee. Indeed, in order to understand the potential variation in the amount of CQAs consumed by coffee drinkers and to go deeper into the influence of brewing techniques on concentration of phenolic compounds, various commercial coffee brews (capsule, pod, instant, iced coffee, and iced cappuccino) were assayed for their CQAs content.

## 2. Material and Methods

### 2.1. Reagents and Standards

**Figure 1:**
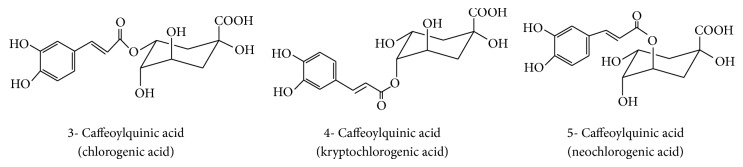
Chemical structures of caffeoylquinic acids studied in the present work [3, 14].

Referenced standard of 5-caffeoylquinic acid (CAS: 906-33-2; purity of 95%) was purchased from Cymit (Barcelona, Spain). Individual standards of 4-caffeoylquinic acid (CAS: 905-99-7; purity of 98%) and of 3-caffeoylquinic acid (CAS: 327-97-9; purity of 95%) were acquired from Sigma-Aldrich (MO, USA). The chemical structures of the main CQAs analyzed in the present study are shown in Figure 1. Solvents were acetonitrile and methanol (HPLC gradient grade) and were obtained from VWR (Belgium). Citric acid and glacial acetic acid with purity of 99% were supplied from Merck (Germany). Zinc acetate dihydrate (purity of 99%) and potassium hexacyanoferrate II trihydrate (purity of 98%) were acquired from VWR (Belgium).

### 2.2. Samples

Twenty-four coffee brews were tested for their CQAs content as follows: eight classical brews (boiled, French, filter, and mocha) prepared using Arabica and Robusta coffee as well as sixteen different commercial samples. A description with regard to the coffees used for brew preparation was exhibited in Table 1.

Roasted Arabica (100%* Coffea arabica*, 2.34% water content) and Robusta (100%* Coffea robusta*, 3.11% water content) coffee, packed in nitrogen-based protective atmosphere, were kindly supplied by a local company of Porto, Portugal. Samples were transported to the lab and kept at −20°C until analysis. Roasted beans were ground by means of a home grinder (Braun KSM 2 model 4041, Mexico). In order to determine the particle size, 50 g of ground coffee was sieved by means of three laboratory test sieves (Retsch, Germany) with different mesh size (212, 300, and 500 *μ*m). Then the particles of each sieve were weighted and presented as percentage from the total mass. Ground Arabica (particle size: 51% >500 *μ*m; 24% >300 *μ*m and <500 *μ*m; 13% >212 *μ*m and <300 *μ*m; 11% <212 *μ*m) and Robusta coffee (particle size: 48% >500 *μ*m; 27% >300 *μ*m and <500 *μ*m; 17% >212 *μ*m and <300 *μ*m; 6% <212 *μ*m) have almost the same particles size distribution. Therefore, the influence of particle size on the extraction of CQAs in both species is almost in the same manner. These ground coffees were used to prepare classical coffee brews (boiled, French, filter, and mocha). Different grind sizes were used for espresso lab made preparation. Arabica coffee beans were ground before brewing by means of La Cimbali, grinder-doser 6/SA. In order to prepare a high quality espresso coffee, a range of particle size from course to very fine ground is advised (particle size: 2% >500 *μ*m; 72% >300 *μ*m and <500 *μ*m; 22% >150 *μ*m and <300 *μ*m; 2% <150 *μ*m and >63 *μ*m).

Various brands of different types of coffee were also purchased randomly from local commerce in Porto, Portugal. Iced coffee and iced cappuccino were supplied by a company from Colombia.

### 2.3. Coffee Brews Preparation

The purpose of the sampling scheme was to comprehensively evaluate the CQAs concentration in a wide range of coffee brews commonly consumed. A total of twenty-four coffee brews were prepared according to the manufacturers' instructions; however, in some cases, information about the coffee origins and species, or roasting conditions used to prepare the blends, was not available. Coffee brews (three replicates for each sample) were stored at −22°C in polypropylene containers until analysis, made in duplicate.

#### 2.3.1. Brews Prepared Using Roasted and Ground Arabica and Robusta Coffee

Nine different coffee samples were obtained using pure Arabica and Robusta coffee with coffee/water ratio of 7.5 g/100 mL to uniformize the comparison of brewing techniques in terms of CQAs content. An exception was homemade espresso coffee which was brewed using Arabica coffee with coffee/water ratio of 7.5 g/40 mL. The preparation modes were as follows.

*Boiled Coffee*. This was prepared by boiling 11.25 g ground coffee with 150 mL of distilled water for 10 min followed by 2 min of settling time followed by decanting the liquid. Individual cup size was 150 mL.

*French Press Coffee*. This was brewed by pouring 150 mL of boiling water onto 11.25 g of ground coffee in glass French press pot followed by stirring. After 2.5 min, the coffee brew was separated from ground coffee by pressing the plunger. Individual cup size was 150 mL.

*Mocha Coffee*. This was brewed using an aluminum mocha pot. Around 11.25 g ground coffee was placed in filter cup. Mocha pot was filled with 150 mL of cold distilled water. The pot was heated until the water reservoir was empty. Individual cup size was 60 mL.

*Filter Coffee*. 22.5 g of roasted and ground coffee was put in a paper filter bag (N° 2) and extracted with 300 mL of boiled distilled water by means of conventional percolation coffee machine KRUPS Aroma Café 5 (Germany). The brew dripped into a heated pot within 2-3 min. The individual cup size was 150 mL. 

*Espresso Coffee.* This was prepared using 7.5 g of finely roasted and ground Arabica coffee using a semiautomatic espresso machine (La Cimbali M31 Classic) with hot water (90 ± 2°C, temperature of water at the exit of the heating unit) under pressure (9.0 ± 0.2 bar) during 21 ± 3 s until the volume in the cup met 40 mL.

#### 2.3.2. Commercial Coffee Brews

Fifteen commercial coffee brews were prepared accordingly to the manufacturers' instructions as follows.

*Capsule Coffee*. Extraction of each capsule was performed using an automatic coffee maker (KRUPS, XN2100, Germany) at a pressure of 19 bar by hot water (93 ± 2°C). All capsules consisted of a plastic cylinder covered by an aluminium film. Amount of coffee in each capsule was as follows: A-type 1 (6.01 ± 0.01 g), A-type 2 (5.01 ± 0.06 g), A-type 3 (5.01 ± 0.03 g), A-type 4 (5.14 ± 0.02 g), A-type 5 (6.13 ± 0.11 g), B (5.19 ± 0.11 g), and C (5.71 ± 0.02 g). Each cup contained 40 mL of coffee brew.

*Pod Espresso*. This was brewed using the SGL coffee machine, designed for pod. The size of a single serving was 40 mL derived from the brewing of a 7.08 ± 0.15 g roasted and ground coffee.

*Instant Coffee*. For this purpose, 2 g of commercial instant coffee powder was extracted with 150 mL of boiled distilled water. Regarding instant espresso, one pack containing 1.8 g of soluble coffee was dissolved in 50 mL of boiled distilled water.

*Iced Coffee*. This was prepared based on preparation instruction where 2 tablespoons of iced coffee powder (8 g) were put in a glass and 240 mL of cold distilled water was added and stirred well.

*Iced Cappuccino*. This was prepared based on preparation instruction as one pack containing 18 g cappuccino powder was put in the glass and 100 mL of cold distilled water was added and stirred well.

*Vending Coffee*. This was obtained from Necta Coffee Vending Machine (Necta Astro Double Brew) to draw a cup of coffee (30 mL).

### 2.4. Sample Extraction and Cleanup

Carrez solutions I (21.9 g of zinc acetate and 3 mL of glacial acetic acid diluted to 100 mL distilled water) and II (10.6 g of potassium hexacyanoferrate II dissolved in 100 mL of distilled water) [15] were used for precipitation of proteins and other interfering compounds as well as the elimination of turbidity and for breaking of the emulsion. Prior to extraction, three cups of each type of brew were defrosted, mixed, and heated to reach a homogeneous mixture at 40–45°C. Extraction of CQAs was performed in duplicate according to the method of Fujioka and Shibamoto [14] with minor modifications. For this purpose, 3.0 mL of coffee was transferred to a polyethylene test tube and treated with 0.1 mL of each Carrez solution (I and II) and 0.8 mL of methanol and the volume was made up with distilled water to 8.0 mL. After dilution, the solution contains 10% methanol. The mixture was vortexed for 1 min and left to stand for 10 min. After centrifugation (Rotofix 32A, Germany) at 4000 rpm for 10 min, the upper phase was filtered through the 0.2 *μ*m PTFE filter membrane (VWR, USA) just before analysis with HPLC-DAD at 325 nm. After the precipitation of the interfering compounds, the average volume of final solution was considered 7.5 mL; therefore, the concentration of CQAs was calculated after applying the dilution factor of 2.5.

### 2.5. Chromatographic Conditions

The instrumental analysis of CQAs was performed using HPLC-DAD, Merck Hitachi Elite La Chromatograph (Tokyo, Japan) equipped with a quaternary system of pumping (L-2130) and L-2455 UV/vis spectrophotometry diode array detector. Separation was achieved using LiChroCART RP-18 endcapped (250 × 4 mm, 5 *μ*m) column, attached to a guard column (4 × 4 mm, 5 *μ*m) of the same kind.

Quantitative analysis of chlorogenic acids was performed based on the method described previously by Tfouni et al. [15] with slight modifications. The mobile phase was constituted eluent A: 10 mM citric acid aqueous solution (pH of 2.4) and eluent B: acetonitrile. The gradient was programmed as follows: from 0 to 30 min 8% of B, 30 to 35 min increase to 80% of B, 35 to 40 min 80% of B, 40 to 45 min decrease to 8% of B, and 45 to 50 min 8% of B. Injected volume was 10 *μ*L and the flow rate of analysis was 1 mL/min. Detection of CQAs was carried out at 325 nm. Identification of the target compounds was confirmed by retention time and spectrum comparison with standard solutions.

### 2.6. Statistical Analysis

To evaluate differences in variation between coffee samples in each class of brewing and also to study the differences among Arabica and Robusta coffee brews, one-way ANOVA was performed with a level of significance of 95%. Data are reported as mean ± standard deviations of two extractions followed by two injections. All statistical analysis was carried out by Minitab 17 software. Graphs were plotted using Microsoft Excel 2007.

## 3. Results and Discussions

### 3.1. Method Validation

Under the experimental conditions referred to above, separation of CQAs could be achieved during the first 30 min with isocratic elution of water (pH: 2.4)/acetonitrile. However, gradient elution was applied to clean the column and remove other interfering compounds for starting the next run. A stock solution containing all CQAs was prepared in aqueous solution of methanol (10%, v/v). Calibration curves were prepared by plotting the peak area against the corresponding concentrations by duplicate injection of 10 *μ*L of standard solutions at nine different concentration levels for 3-CQA (2–400 mg/L), 4-CQA (1–200 mg/L), and 5-CQA (1–200 mg/L).

Regarding the detector response, the regression lines were linear over the studied concentration range and the corresponding coefficients of correlation (*R* ^2^) of 0.999 were obtained for all analyzed compounds. The sensitivity of the method, expressed as the slope of the calibration curve, was maximum for 4-CQA. The limit of detection (LOD) and limit of quantification (LOQ) were calculated at signal to noise ratio of three (*S*/*N* = 3) and ten (*S*/*N* = 10), respectively. The LODs were 0.37, 0.39, and 0.18 mg/L for 3-CQA, 4-CQA, and 5-CQA, respectively. For LOQs, 1.24 mg/L was obtained for 3-CQA and 1.29 mg/L and 0.58 mg/L were achieved for 4-CQA and 5-CQA, respectively. Repeatability of the method (intraday precision) was estimated when the CQAs standards at three concentration levels (C1, C2, and C3, see the concentrations in Table 2) were analyzed on the same day for six injections. Reproducibility (interday precision) was the result of the analysis of standards at three concentration levels (C1, C2, and C3; see the concentrations in Table 2) during the three sequential days by injecting three times and the average %CV was reported in Table 2.

Intraday precision and recovery of CQAs in some coffee brews, spiked at two different concentration levels, were exhibited in Table 3. The recovery test was performed by spiking various types of coffee brews with known quantity of the CQAs reference standards before the extraction procedure. The fortified sample was then extracted and analyzed in triplicate as described previously. The average recovery (%) was reported as the mean ratio between the obtained and the expected concentration of CQAs in fortified samples. Different coffee brews were selected based on their initial CQAs concentration (filter, instant, and capsule coffee) so after spiking, the concentration of CQAs in spiked samples was within the linearity range. The mean recoveries ranged between 91.46% and 103.39% (Table 3).

### 3.2. CQAs Content in Coffee Brews

In the present study, samples were divided into two groups. Firstly, the effects of brewing procedures as well as the effect of coffee species (Arabica and Robusta) on CQAs content of classical brewing techniques were evaluated and afterwards commercial coffee brews including capsule, pod, instant, iced coffee, and iced cappuccino were compared with regard to their CQAs concentration.

#### 3.2.1. Brews Prepared Using Roasted and Ground Arabica and Robusta Coffee

Chlorogenic acids content in brews prepared using roasted and ground Arabica and Robusta coffee including boiled, French, mocha, and filter coffee are shown in Table 4. Our findings revealed the occurrence of high concentration of CQAs in all studied samples. As it can be clearly seen in Table 4, the major isomer in these classes of samples was 3-CQA, accounting for about 50% of the total CQAs, followed by 5-CQA and 4-CQA, accounting for about 25-26% for each one, both for Arabica and Robusta coffee. During the extraction, most of the water extractable components are extracted at the beginning of the extraction process [19] but lower concentration of 5-CQA than 3-CQA could be explained by the fact that 5-CQA is less water-soluble than 3-CQA, yielding lower concentration in the brews [16]. Although some bibliographic references reveal 5-CQA as the main isomer among CQAs [10, 14, 18] our results were comparable with the ones obtained by Gloess et al. [16], who found 3-CQA at higher concentration in various types of coffee brews. Crozier et al. [6] proved that during roasting, 3-CQA and 4-CQA are destroyed more slowly than 5-CQA. In another study, Farah et al. [11] found the reduction of 5-CQA from green beans to light roasted beans while 3-CQA and 4-CQA content increased in light roasted beans and then gradually decreased at higher roasted degree. Therefore, due to the different sensitivity of CQAs to various roasting conditions [11, 12], the higher concentration of 3-CQA than 4- or 5-CQA might be explained by the origin of the beans and their roasting degree, which is however unknown for us.

In both species, when considering the brewing procedure, the mocha extraction was the most efficient brewing method followed by boiled, French, and filter coffee. Since these samples were prepared with coffee/water ratio of 7.5 g/100 mL, the effect of this parameter on CQAs content could be eliminated. Besides that, ground Arabica and Robusta coffee have almost the similar particle size distribution so the degree of grinding seems to have similar effect on CQAs content. The most influencing parameters seem to be extraction temperature and pressure because mocha extraction was performed under pressure (0.5 relative atmospheres, corresponding to 110°C) [20]. The decreasing order of total CQAs of samples prepared with Robusta coffee was mocha (872.93 mg/L) > boiled (771.29 mg/L) > French (666.67 mg/L) > filter coffee (624.03 mg/L). Regarding the Arabica species, the decreasing order was similar to Robusta, although the total concentrations of CQAs in mocha (744.04 mg/L) and boiled coffee (744.70 mg/L) were almost the same (*P* > 0.05) followed by French (645.56 mg/L) and filter coffee (638.58 mg/L). In the present study, filtered brews are the ones that least contribute to CQAs intake and provide the lowest content of CQAs. Indeed, despite the other studied brewing techniques, during the filter coffee brew preparation, ground coffee was only washed out with hot water at ambient pressure without any flotation, therefore yielding lower CQAs contents than other brewed coffees.

Tfouni et al. [15] also found the higher content of CQAs in boiled coffee (26–295 mg/100 mL) than filter coffee (24–219 mg/100 mL). This could be due to the higher contact time between ground coffee and hot water during the boiled extraction procedure [15]. In previous work, Pérez-Martínez et al. [21] observed that mocha coffee was the richest source of CGAs, followed by the filter and plunger coffee makers. Concerning the CQAs content of French press, results were opposite to Gloess et al. [16] who indicated higher extraction efficiency of 3-CQA and 5-CQA in French press than in mocha or even espresso coffee. This difference could be explained by different coffee/water ratio and extraction time that they used for mocha and French press brew preparations.

Considering the influence of the raw material, in general, the CQAs content of different coffee brews was significantly (*P* < 0.05) affected by the coffee species, as Robusta samples yielded greater CQAs content than the Arabica ones. Levels ranging from 624.03 to 872.93 mg/L for Robusta and from 638.58 mg/L to 744.70 mg/L for Arabica were detected in analyzed coffee brews (Table 4). The obtained results were in accordance with Tfouni et al. [15] where Robusta coffee brews contain higher CQAs than the Arabica ones. The biggest difference was found among mocha coffees with concentration of 872.93 mg CQAs/L for Robusta and 744.04 mg CQAs/L for Arabica. Exception was filter coffee, where there was no remarkable difference between the values of total CQAs for Arabica (95.79 mg/L) and for Robusta (93.60 mg/L) (*P* > 0.05). There was an agreement with the results obtained by Ludwig et al. [19] regarding the sum of 3-, 4-, and 5-CQAs in Arabica filter coffee (81.0 mg/100 mL) which was higher than Robusta filter coffee (56.2 mg/100 mL). Similar behaviour of Arabica and Robusta coffee at different roasting degree was also reported previously [22].

Some authors attribute the lower concentration of CGAs in Arabica than in Robusta to the coffee production step (wet or dry method). Generally, wet method is used for Arabica coffee and requires substantial amounts of water. It could be a reason for loss of CGAs in Arabica coffee in comparison to the Robusta coffee that is commonly processed by the dry method [23]. According to Leloup et al. [24] and Clifford [25], although green Robusta beans have a higher CGAs content, the sensitivity of CGAs in Robusta coffee matrix seems to be more than that in Arabica coffee matrix which could explain the same behaviour of Arabica and Robusta coffee brews in some cases.

**Figure 2:**
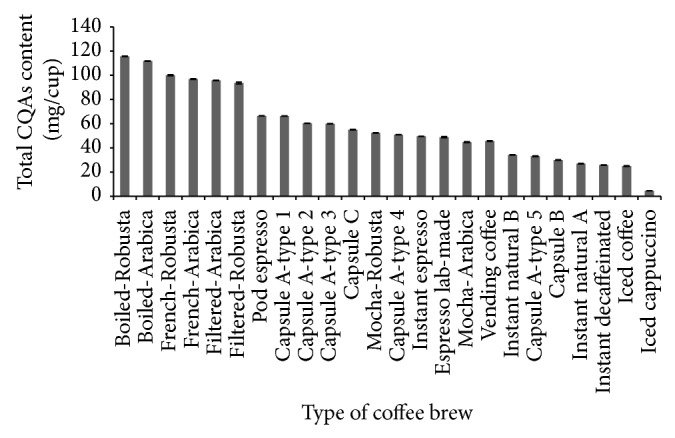
Total CQAs content of various types of coffee brews per cup. Cup sizes were boiled (150 mL), French (150 mL), mocha (60 mL), filter (150 mL), capsules, pod, espresso lab-made (40 mL), vending coffee (30 mL), instant espresso (50 mL), natural and decaffeinated instant coffees (150 mL), ice coffee (240 mL), and iced cappuccino (100 mL).

Although, based on the concentration basis (mg/L), mocha produced a high concentrated brew than others in terms of CQAs, that finding is different when content per cup size is considered. As it can be seen in Figure 2, boiled coffee has the greatest amount of CQAs per cup (115.69 and 111.71 mg/150 mL in Robusta and Arabica, resp.), and mocha has the less content both in Robusta (52.38 mg/60 mL) and Arabica coffee (44.64 mg/60 mL). It means that consumption of a cup of boiled coffee contributes to higher intake of CQAs by consumers followed by French, filter, and mocha.

It should be mentioned that espresso lab-made prepared with roasted and ground Arabica coffee was compared with commercial brews prepared under pressure.

#### 3.2.2. Commercial Coffee Brews

In order to understand the variation in the amount of CQAs consumed by coffee drinkers and to go deeper into the influence of brewing techniques on the concentration of phenolic compounds, various commercial coffee brews were assayed in this section. Indeed, analysis of commercial coffee brews is of interest because they are representative of real samples which are delivered outside the laboratory conditions. As previously mentioned, comparison of commercial coffee brews was more complicated due to the lack of information regarding ratio of each species in the blend, roasting conditions, grinding degree, and the origin of the beans used for brewing.

The results of CQAs concentration in different commercial coffee brews expressed as mg/L are displayed in Table 4. Analysis of the coffee samples indicates the presence of 3-, 4-, and 5-CQA in all samples. The most abundant CQAs in all considered samples (except instant natural A) was 3-CQA accounting for 34–50% of the total CQAs followed by 23–36% for 5-CQA and 25 to 28% for 4-CQA. Generally speaking, the results of the processes studied varied according to the brewing mechanisms and total CQAs ranged from 45.79 mg/L in iced cappuccino to 1662.01 mg/L in pod espresso.

Regarding the CQAs content of capsules, the results were in the range of 748.40 to 1656.82 mg CQAs/L, much higher than those reported for classical brew preparation. Capsule A-type 1 was found to produce the higher concentrated brew in terms of total CQAs (1656.82 mg/L). Since all capsules were brewed with the same machine and conditions, the effect of water temperature and pressure on CQAs contents would be similar. Information of the label of the product revealed that capsule A-type 1 is a blend of Arabica and Robusta from different origins which were ground finely; however, the ratios between species are unlikely to be identical and both are known to influence the CQAs content [26]. Frequently, among capsules A, the lowest CQAs content was reported in capsule A-type 5 that has the highest roasting intensity, which may possess more degradation of CQAs during the roasting. Although the coffee quantity of capsule A-type 5 (6.13 ± 0.11 mg/capsule) was almost similar to A-type 1 (6.01 ± 0.01 g/capsule), their country of origins and coffee variety may be different which could explain the diversity of CQAs among these two types of capsules from the same brand. Among all analyzed capsule coffee, capsule B had less quantity of coffee powder (5.19 ± 0.11 g/capsule) which may explain its less concentration of CQAs (748.40 mg CQAs/L) than other capsules.

There are limited studies regarding CQAs content in capsule coffee [16, 17]. Gloess et al. [16] observed 3-CQA in concentration of 15 mg/30 mL and found 5-CQA in lower concentration (6 mg/30 mL) in Nespresso coffee variety of “Arpeggio.”

According to the results of HPLC analysis, pod espresso revealed the greatest content of CQAs (1662.01 mg/L), corresponding to 823.45, 436.30, and 402.30 mg/L for 3-CQA, 4-CQA, and 5-CQA, respectively. These high concentrations could be attributed to the high quantity of coffee per pod (7.08 ± 0.15 g/pod). Grinding degree, ratio of Arabica to Robusta in the blend of coffee pod, could also influence the extraction of CQAs, together with other technological factors like water pressure, which was unknown.

Another concentrated brew with regard to CQAs seems to be vending coffee (1521.05 mg/L) and espresso lab-made (1220.35 mg/L). In the case of espresso lab-made, the variety of coffee (100% Arabica) may play an important role in CQAs content.

The results obtained in the present study confirmed the presence of high concentration of CQAs in various espresso coffee (capsules, pod, or normal espresso) ranging from 748.40 to 1662.01 mg/L. However, Caprioli et al. [18] reported the CQAs content up to 2223.4 mg/L in different espresso coffee. The presence of CQAs in various types of espresso coffee was also affirmed by Niseteo et al. [27] who obtained 495.56–985.73 mg CQAs/L, which is in compliance with the CQAs content determined in the present study (748.40–1662.01 mg/L).

Although espresso coffees (capsules, pod,or normal espresso) contain high concentration of CQAs, their average content per cup was found to be less than classical techniques such as boiled, French, and filter coffee (Figure 2). The total CQAs content per cup ranging between 29.94 and 66.27 mg/40 mL was found in brewed coffee using capsule B and pod espresso, respectively. Gloess et al. [16] presented 3-CQA and 5-CQA in the levels of 18 and 8 mg/30 mL of espresso coffee prepared with semiautomatic machine, respectively.

In the case of instant brewing technique, despite the other brews, the main isomer in instant natural A was 5-CQA (36%) followed by 3-CQA (34%) and 4-CQA (28%). Accounting for the total CQAs, the greatest amount was obtained for instant espresso (991.85 mg/L) followed by instant natural B (227.57 mg/L), natural A (179.16 mg/L), and instant decaffeinated (171.86 mg/L). These values are in accordance with some authors which developed a study for comparison of normal coffee over decaffeinated coffee [4, 14, 27] and loss of CGAs in decaffeinated coffee was reported. However, it must be taken into account that soluble coffee suffers an additional thermal extraction treatment at high temperature after roasting which decreased their antioxidant capacity [28]. These additional processes may affect the CQAs content due to the interaction of CGAs with Maillard reaction intermediates [10].

These data demonstrate that when comparing commercial soluble coffee as mg/cup, they could be accounted as the potential source for delivery of moderate level of CQAs as instant espresso delivered around 50 mg CQAs per cup of 50 mL. Mills et al. [10] reported the CGAs ranging from 37.04 to 121.25 mg/200 mL in various soluble coffees. The higher content than our study is probably due to the higher consumed cup size (200 mL). Despite our results, Niseteo et al. [27] found the instant coffee as one of the richest sources of CQAs with concentration ranging from 2300.77 to 4034.41 mg/L in various types of soluble coffee.

In general, the lowest concentration of CQAs was found in iced cappuccino (45.79 mg/L, corresponding to 4.58 mg/100 mL) and iced coffee (104.19 mg/L, corresponding to 25.00 mg/240 mL). The presence of CQAs in cappuccino prepared with hot water was previously reported by Niseteo et al. [27] in the range of 15.89–104.65 mg/L. It must be taken into account that for iced coffee and iced cappuccino, there are an additional process including adding other ingredients like milk and sugar which will influence the presence of CQAs in final product [27]. According to Narita and Inouye [29] presence of 5-CQA is pH dependent where at lower pH it is more stable and by incubation at 37°C in high pH (7.4, 8.0, 8.5, and 9.0), 3-CQA and 4-CQA were produced from isomerization of 5-CQA. Besides that, total CQAs were decreased gradually at pH of 5.0–9.0 [29]. The effect of milk on antioxidant capacity can be attributed to the precipitation of polyphenols due to the binding with milk proteins such as casein [27, 30]. It is worth noting that although iced coffee and in particular iced cappuccino contain ingredient such as milk which result in high pH beverages and subsequently degradation of CQAs, the less ratio of coffee in these products than other brews which are prepared only from pure coffee powder may also affect the amount of CQAs in final products.

## 4. Conclusions

This investigation clearly demonstrated that coffee brews commonly consumed are capable of delivering high amounts of CQAs as the major isomer in analyzed samples was 3-CQA, followed by 5-CQA and 4-CQA. Besides that it was confirmed that brewing mechanisms have a profound effect on the amount of CQAs delivered per cup. Since chlorogenic acids play an important role in human health, this study allowed us to elucidate the role of brewing techniques and type of coffee on CQAs content of brewed coffee and subsequently allowing us to equilibrate the acidity of brews for consumers. This equilibration lets consumers to avoid consequences of high CGAs consumption and at the same time they intake sufficient amount for medicinal purposes.

## Figures and Tables

**Table 1:** General description of ground coffees used for preparation of various types of coffee brews studied in the present work^a^.

Type of coffee	Description	Roasted condition
Roasted and ground Arabica coffee (used to prepare 4 samples include boiled, French, filter, and mocha coffee)	100% Arabica, 2.34% water content	NA^b^
Roasted and ground Robusta coffee (used to prepare 4 samples include boiled, French, filter, and mocha coffee)	100% Robusta, 3.11% water content	NA
Capsule A—type 1	Blend of Arabica and Robusta	Roasted slowly and fine grinding
Capsule A—type 2	Blend of Arabica and Robusta	Light roasted and fine grinding
Capsule A—type 3	Blend of Arabica and Robusta	Light roasted
Capsule A—type 4	Blend of Arabicas	Light roasted
Capsule A—type 5	Blend of Arabicas	Long roasting at low temperatures
Capsule B	Blend of Arabica and Robusta	Medium roasted
Capsule C	100% Arabica coffee	NA
Vending coffee	NA	NA
Pod espresso	Blend of Arabica and Robusta	NA
Espresso lab-made	100% Arabica, 2.34% water content	NA
Instant natural A	Soluble coffee natural	NA
Instant natural B	Soluble coffee natural	NA
Instant decaffeinated	Soluble coffee decaffeinated	NA
Instant espresso	Blend of Arabicas	NA
Iced coffee	Instant coffee, sugar, acid citric, and so forth	NA
Iced cappuccino	Instant coffee, sugar, skimmed milk powder, and so forth	NA

^a^All information was adopted from the label of coffee products.

^b^Not available.

**Table 2:** Validation parameters for CQAs analysis by HPLC-DAD.

Validation parameters	Concentration levels^a^	3-CQA	4-CQA	5-CQA
Linearity range (mg/L)	—	2–400	1–200	1–200
*R* ^2^ (*N* = 9)^b^	—	0.999	0.999	0.999
Sensitivity^c^	—	116292	122162	111337
Limit of detection (mg/L)^d^	—	0.37	0.39	0.18
Limit of quantification (mg/L)^d^	—	1.24	1.29	0.58
Intraday precision (%CV)	C1	1.06	0.95	0.36
	C2	1.06	0.80	0.54
	C3	0.31	0.17	0.24
Interday precision (%CV)	C1	0.81	0.54	0.36
	C2	0.46	0.68	0.23
	C3	1.11	1.41	0.32

^a^Concentration of each compound in standard solutions was as follows. C1: 3-CQA (40 mg/L), 4-CQA (20 mg/L), and 5-CQA (20 mg/L); C2: 3-CQA (160 mg/L), 4-CQA (80 mg/L), and 5-CQA (80 mg/L); C3: 3-CQA (320 mg/L), 4-CQA (160 mg/L), and 5-CQA (160 mg/L).

^b^ *R* ^2^ coefficient of determination, *N* number of calibration curve standards.

^c^Sensitivity was expressed as the slope of the calibration curves.

^d^Calculated from the signal to noise ratio of 3 (LOD) and 10 (LOQ).

**Table 3:** Intraday precision and recovery of CQAs in coffee brews, spiked at two different concentration levels.

Spiked level^a^	Analyte	Initial concentration (mg/L)	Precision (%CV)	Recovery (%)
Filter coffee (prepared from roasted and ground Arabica coffee)				
C1	3-CQA	307.86	1.50	92.71
	4-CQA	169.72	1.59	98.32
	5-CQA	161.00	1.82	96.91
C2	3-CQA	307.86	1.96	90.38
	4-CQA	169.72	0.86	97.09
	5-CQA	161.00	1.03	97.10

Instant natural A				
C1	3-CQA	62.51	1.77	98.81
	4-CQA	51.26	1.58	100.64
	5-CQA	65.38	1.88	97.06
C2	3-CQA	62.51	1.08	91.46
	4-CQA	51.26	2.00	94.80
	5-CQA	65.38	0.53	93.90

Capsule coffee (A—type 5)				
C1	3-CQA	369.27	2.21	101.89
	4-CQA	234.95	2.17	102.51
	5-CQA	222.08	2.24	100.00
C2	3-CQA	369.27	1.57	99.83
	4-CQA	234.95	1.65	101.31
	5-CQA	222.08	1.74	103.39

^a^Spiked samples were prepared at two concentrations levels as follows. C1: 3-CQA (80 mg/L), 4-CQA (40 mg/L), and 5-CQA (40 mg/L); C2: 3-CQA (240 mg/L), 4-CQA (120 mg/L), and 5-CQA (120 mg/L).

**Table 4:** Caffeoylquinic acids (CQAs) content in various types of coffee brews^a^.

Class of coffee brews	3-CQA	4-CQA	5-CQA	Total CQAs^b^	As a percentage of total CQA (%)		
	(mg/L)	(mg/L)	(mg/L)	(mg/L)	3-CQA	4-CQA	5-CQA
Classical coffee brews							
Brews prepared using roasted and ground Arabica coffee							
Boiled	352.57 ± 1.64	197.79 ± 2.07	194.34 ± 0.99	744.70 ± 0.54^a^	47.34	26.56	26.10
French	310.97 ± 4.05	171.48 ± 1.17	163.12 ± 0.99	645.56 ± 1.71^b^	48.17	26.56	25.27
Mocha	357.25 ± 15.59	198.47 ± 8.31	188.32 ± 6.30	744.04 ± 4.89^a^	48.02	26.67	25.31
Filter	307.86 ± 1.97	169.72 ± 0.46	161.00 ± 1.21	638.58 ± 0.75^b^	48.21	26.58	25.21
Brews prepared using roasted and ground Robusta coffee							
Boiled	365.44 ± 6.70	199.67 ± 3.88	206.18 ± 3.59	771.29 ± 1.72^b^	47.38	25.89	26.73
French	320.35 ± 6.94	172.85 ± 1.88	173.47 ± 2.11	666.67 ± 2.86^c^	48.05	25.93	26.02
Mocha	421.49 ± 7.95	225.47 ± 2.85	225.97 ± 2.61	872.93 ± 3.02^a^	48.28	25.83	25.89
Filter	296.83 ± 12.60	162.02 ± 3.78	165.17 ± 3.31	624.03 ± 5.23^d^	47.57	25.96	26.47
Commercial coffee brews							
Capsule coffees							
Capsule A—type 1	818.93 ± 4.22	444.69 ± 4.78	393.20 ± 4.16	1656.82 ± 0.34^a^	49.43	26.84	23.73
Capsule A—type 2	710.01 ± 3.77	420.11 ± 1.85	378.94 ± 0.66	1509.06 ± 1.57^b^	47.05	27.84	25.11
Capsule A—type 3	720.50 ± 0.61	408.77 ± 4.35	370.35 ± 2.11	1499.63 ± 1.88^c^	48.05	27.26	24.70
Capsule A—type 4	604.36 ± 5.05	349.22 ± 2.43	318.57 ± 1.19	1272.15 ± 1.97^e^	47.51	27.45	25.04
Capsule A—type 5	369.27 ± 13.98	234.95 ± 12.49	222.08 ± 1.80	826.29 ± 6.65^f^	44.69	28.43	26.88
Capsule B	356.74 ± 11.87	200.84 ± 1.15	190.8 ± 1.30	748.40 ± 6.14^g^	47.67	26.84	25.50
Capsule C	688.95 ± 12.36	362.20 ± 4.86	323.64 ± 6.15	1374.78 ± 4.61^d^	50.11	26.35	23.54
Other pressure methods							
Vending coffee	713.64 ± 21.24	398.39 ± 3.57	409.02 ± 3.61	1521.05 ± 10.19^b^	46.92	26.19	26.89
Pod espresso	823.45 ± 9.82	436.30 ± 5.64	402.30 ± 3.55	1662.01 ± 3.19^a^	49.55	26.25	24.20
Espresso lab-made	551.15 ± 27.79	337.07 ± 14.93	332.13 ± 1.05	1220.35 ± 13.37^c^	45.16	27.62	27.22
Instant coffees							
Instant natural A	62.51 ± 2.51	51.26 ± 0.34	65.38 ± 1.15	179.16 ± 1.10^c^	34.89	28.61	36.50
Instant natural B	100.22 ± 0.78	58.56 ± 2.13	68.80 ± 0.40	227.57 ± 3.31^b^	44.04	25.73	30.23
Instant decaffeinated	62.78 ± 2.87	48.53 ± 2.58	60.55 ± 0.54	171.86 ± 1.27^c^	36.53	28.24	35.23
Instant espresso	412.07 ± 5.26	278.46 ± 3.26	301.32 ± 2.88	991.85 ± 1.28^a^	41.55	28.07	30.38
Other brews							
Iced coffee	44.51 ± 5.21	27.82 ± 3.38	31.85 ± 3.50	104.19 ± 1.02^b^	42.72	26.71	30.57
Iced cappuccino	17.01 ± 0.43	12.57 ± 0.39	16.21 ± 0.49	45.79 ± 0.05^a^	37.14	27.46	35.40

^a^The results correspond to the average ± standard deviation of two extractions followed by two times injection.

^b^In each class of brew, values with the same letter are not significantly different (*P* > 0.05).

## References

[1] George S. E., Ramalakshmi K., Rao L. J. M. (2008). A perception on health benefits of coffee. *Critical Reviews in Food Science and Nutrition*.

[2] Farah A., Donangelo C. M. (2006). Phenolic compounds in coffee. *Brazilian Journal of Plant Physiology*.

[3] Ma Y.-C., Wang X.-Q., Hou F., Ma J., Luo M., Chen A., Jin P., Lu S., Xu I. (2011). Rapid resolution liquid chromatography (RRLC) analysis and studies on the stability of Shuang-Huang-Lian preparations. *Journal of Pharmaceutical and Biomedical Analysis*.

[4] Farah A., de Paulis T., Moreira D. P., Trugo L. C., Martin P. R. (2006). Chlorogenic acids and lactones in regular and water-decaffeinated arabica coffees. *Journal of Agricultural and Food Chemistry*.

[5] Rodrigues N. P., Bragagnolo N. (2013). Identification and quantification of bioactive compounds in coffee brews by HPLC-DAD-MS*^n^*. *Journal of Food Composition and Analysis*.

[6] Crozier T. W. M., Stalmach A., Lean M. E. J., Crozier A. (2012). Espresso coffees, caffeine and chlorogenic acid intake: potential health implications. *Food & Function*.

[7] Xiang Z., Ning Z. (2008). Scavenging and antioxidant properties of compound derived from chlorogenic acid in South-China honeysuckle. *LWT: Food Science and Technology*.

[8] Sun Z. X., Liu S., Zhao Z. Q., Su R. Q. (2014). Protective effect of chlorogenic acid against carbon tetrachloride-induced acute liver damage in rats. *Chinese Herbal Medicines*.

[9] Kim J., Lee S., Shim J., Kim H. W., Jang Y. J., Yang H., Park J., Choi S. H., Yoon J. H., Lee K. W., Lee H. J. (2012). Caffeinated coffee, decaffeinated coffee, and the phenolic phytochemical chlorogenic acid up-regulate NQO1 expression and prevent H_2_O_2_-induced apoptosis in primary cortical neurons. *Neurochemistry International*.

[10] Mills C. E., Oruna-Concha M. J., Mottram D. S., Gibson G. R., Spencer J. P. E. (2013). The effect of processing on chlorogenic acid content of commercially available coffee. *Food Chemistry*.

[11] Farah A., de Paulis T., Trugo L. C., Martin P. R. (2005). Effect of roasting on the formation of chlorogenic acid lactones in coffee. *Journal of Agricultural and Food Chemistry*.

[12] Moon J.-K., Hyui Yoo S. U. N., Shibamoto T. (2009). Role of roasting conditions in the level of chlorogenic acid content in coffee beans: correlation with coffee acidity. *Journal of Agricultural and Food Chemistry*.

[13] Campa C., Doulbeau S., Dussert S., Hamon S., Noirot M. (2005). Qualitative relationship between caffeine and chlorogenic acid contents among wild *Coffea* species. *Food Chemistry*.

[14] Fujioka K., Shibamoto T. (2008). Chlorogenic acid and caffeine contents in various commercial brewed coffees. *Food Chemistry*.

[15] Tfouni S. A. V., Carreiro L. B., Teles C. R. A., Furlani R. P. Z., Cipolli K. M. V. A. B., Camargo M. C. R. (2014). Caffeine and chlorogenic acids intake from coffee brew: influence of roasting degree and brewing procedure. *International Journal of Food Science & Technology*.

[16] Gloess A. N., Schönbächler B., Klopprogge B., D'Ambrosio L., Chatelain K., Bongartz A., Strittmatter A., Rast M., Yeretzian C. (2013). Comparison of nine common coffee extraction methods: instrumental and sensory analysis. *European Food Research and Technology*.

[17] Parenti A., Guerrini L., Masella P., Spinelli S., Calamai L., Spugnoli P. (2014). Comparison of espresso coffee brewing techniques. *Journal of Food Engineering*.

[18] Caprioli G., Cortese M., Odello L., Ricciutelli M., Sagratini G., Tomassoni G., Torregiani E., Vittori S. (2013). Importance of espresso coffee machine parameters on the extraction of chlorogenic acids in a certified Italian espresso by using SPE-HPLC-DAD. *Journal of Food Research*.

[19] Ludwig I. A., Sanchez L., Caemmerer B., Kroh L. W., de Peña M. P., Cid C. (2012). Extraction of coffee antioxidants: impact of brewing time and method. *Food Research International*.

[20] Petracco M., Clarke R. J., Vitzthum O. G. (2001). Technology IV: beverage preparation, brewing trends for the new millennium. *Coffee: Recent Developments*.

[21] Pérez-Martínez M., Caemmerer B., De Peña M. P., Concepción C., Kroh L. W. (2010). Influence of brewing method and acidity regulators on the antioxidant capacity of coffee brews. *Journal of Agricultural and Food Chemistry*.

[22] Trugo L. C., Macrae R. (1984). Chlorogenic acid composition of instant coffees. *The Analyst*.

[23] Duarte G. S., Pereira A. A., Farah A. (2010). Chlorogenic acids and other relevant compounds in Brazilian coffees processed by semi-dry and wet post-harvesting methods. *Food Chemistry*.

[24] Leloup V., Gancel C., Liardon R., Rytz A., Pithon A. Impact of wet and dry process on green composition and sensory characteristics.

[25] Clifford M. N. The nature of chlorogenic acids. Are they advantageous compounds in coffee.

[26] Andueza S., de Peña M. P., Cid C. (2003). Chemical and sensorial characteristics of espresso coffee as affected by grinding and torrefacto roast. *Journal of Agricultural and Food Chemistry*.

[27] Niseteo T., Komes D., Belščak-Cvitanović A., Horžić D., Budeč M. (2012). Bioactive composition and antioxidant potential of different commonly consumed coffee brews affected by their preparation technique and milk addition. *Food Chemistry*.

[28] Vignoli J. A., Bassoli D. G., Benassi M. T. (2011). Antioxidant activity, polyphenols, caffeine and melanoidins in soluble coffee: the influence of processing conditions and raw material. *Food Chemistry*.

[29] Narita Y., Inouye K. (2013). Degradation kinetics of chlorogenic acid at various pH values and effects of ascorbic acid and epigallocatechin gallate on its stability under alkaline conditions. *Journal of Agricultural and Food Chemistry*.

[30] Sharma V., Vijay Kumar H., Rao L. J. M. (2008). Influence of milk and sugar on antioxidant potential of black tea. *Food Research International*.

